# Supplementary material for: Content-rich biological network constructed by mining PubMed abstracts
Source: BMC Bioinformatics. 2004 Oct 8;5:147. doi: 10.1186/1471-2105-5-147 (PMC528731; doi:10.1186/1471-2105-5-147)
Supplement: Additional File 2 — The original results of the above study (non-essential files are deleted to keep the file size under the limit set by BMC bioinformatics). [file 1471-2105-5-147-S2.bz2 › chilibotAdditionalFile2/dip20/303ID9501253E1300/html/APP_A2M.html]

 


 **APP** and **A2M** 
  
Found 50 abstracts in PubMed, retrieved 20.  
 

 What does Google say? 
 PDF only 
| .edu only 

---

**Interactive relationship** (e.g. stimulation, inhibition, etc)

**Inhibitory relationship**- Alpha 2 macroglobulin  [ **A2M** ]   **A2M**  is a proteinase inhibitor that is present in senile plaques and may play a role in metabolism of amyloid beta  [ **APP** ]  A beta peptide.  Ref: 10477119 Neurosci Lett, 1999
- Alpha 2 macroglobulin  [ **A2M** ]   **A2M**  is a serum pan protease inhibitor that is related with the pathogenesis of Alzheimer s disease AD through its ability to mediate amyloid beta  [ **APP** ]  degradation.  Ref: 10936700 Neurosci Lett, 2000
- Alpha 2 macroglobulin  [ **A2M** ]  encoded by the gene  **A2M**  is a serum pan protease inhibitor that may be related with the pathogenesis of Alzheimer s disease AD because of its ability to mediate amyloid beta  [ **APP** ]  degradation.  Ref: 10477120 Neurosci Lett, 1999
**Neutral relationship**- Haptoglobin Hp and alpha 2 macroglobulin  [ **A2M** ]  alpha 2 M  [ **A2M** ]  are among the most important  **APP**  in rats.  Ref: 11521461 Vojnosanit Pregl< MedlineTA>Vojnosanit Pregl, 1999
- BACKGROUND alpha2 Macroglobulin  **A2M**  forms the complex with amyloid beta  [ **APP** ]  protein Abeta and is associated with degradation of Abeta.  Ref: 10668711 Neurology, 2000
- BACKGROUND AND PURPOSE alpha 2 Macroglobulin  [ **A2M** ]  may be implicated in amyloid beta  [ **APP** ]  protein deposition.  Ref: 10548657 Stroke, 1999

**Non-interactive relationship** (e.g. studied together, co-existance, homology, etc.)

- Alpha 2 macroglobulin  [ **A2M** ]  mediated degradation of amyloid beta  [ **APP** ]  1 42 a mechanism to enhance amyloid beta  [ **APP** ]  catabolism.  Ref: 11161627 Exp Neurol, 2001
- The responses of liver glucocorticoid receptor GR and genes coding for a glucocorticoid inducible tyrosine aminotransferase TAT and two acute phase proteins  **APP**  alpha2 macroglobulin alpha2  [ **A2M** ]  M and gamma fibrinogen Fb to changes in glucocorticoid GC and proinflammatory AP cytokine contents have been examined in rats after single or combined treatments with turpentine oil, dexamethasone Dex and adrenalectomy.  Ref: 11490095 Biol Signals Recept< MedlineTA>Biol Signals Recept, 2001
- As for the three early onset genes the amyloid beta  [ **APP** ]  protein precursor and the two presenilins and for the other late onset gene ApoE, DNA polymorphisms in the  **A2M**  gene associated with AD result in significantly increased accumulation of amyloid plaques in AD brains.  Ref: 10959035 Exp Gerontol, 2000
- Most of these studies have examined only one or two polymorphisms in a defined population, which provides a lack of a general view of the Alzheimer s disease genetic component.OBJECTIVETo overcome this limitation, nine polymorphisms in seven different candidate genes  **A2M** , ACT, APOE,  **APP** , BH, HSP70 2, and IL1 A were genotyped.METHODSThe sample comprised 112 Alzheimer s disease patients and 89 controls from Spain.  Ref: 12782964 Psychiatr Genet, 2003
- We now demonstrated that the  **APP**  RK transgene did not disturb the expression of several other genes, i.e. endogenous amyloid precursor protein and amyloid precursor protein like proteins, members of the low density lipoprotein receptor lipoprotein receptor family and several of their ligands, including apolipoprotein E, but expression of alpha 2 macroglobulin  [ **A2M** ]  was never detected.  Ref: 10391465 Neuroscience, 1999
- While rare mutations in the early onset genes amyloid precursor protein, and presenilins 1 and 2 lead to increased generation of specific forms of the amyloid beta  [ **APP** ]  protein A, beta, common polymorphisms in the late onset genes apolipoprotein E and alpha 2 macroglobulin  [ **A2M** ]  are thought to alter the clearance and degradation of A, beta in brain.  Ref: 11464541 Rev Med Chil, 2001
- We found that this endocytic receptor conferred protection against amyloid beta  [ **APP** ]  protein toxicity in the presence of activated alpha 2 macroglobulin  [ **A2M** ]  and its down regulation via inhibition by receptor associated protein or transfection of cells with presenilin 1, increased susceptibility to amyloid beta  [ **APP** ]  protein toxicity.  Ref: 10899157 J Biol Chem, 2000
- In order to delineate the neuroprotective role of the low density lipoprotein receptor related protein LRP against amyloid beta  [ **APP** ]  protein toxicity, studies were performed in C6 cells challenged with amyloid beta  [ **APP** ]  protein in the presence or absence of activated alpha 2 macroglobulin  [ **A2M** ] .  Ref: 10899157 J Biol Chem, 2000
